# Are estimates of faces’ ages less accurate when they wear sunglasses or face masks and do these disguises make it harder to later recognise the faces when undisguised?

**DOI:** 10.1186/s41235-022-00370-0

**Published:** 2022-02-16

**Authors:** Craig Thorley, Benjamin Acton, Jesse Armstrong, Shanade Ford, Margaret Gundry

**Affiliations:** Department of Psychology, James Cook University, Townsville, 4814 Australia

**Keywords:** Age perception, Age estimation, Face recognition, Disguises, Sunglasses, Face masks, COVID-19

## Abstract

This study examined whether our ability to accurately estimate unfamiliar faces’ ages declines when they are wearing sunglasses or surgical-style face masks and whether these disguises make it harder to later recognise those faces when undisguised. In theory, both disguises should harm age estimation accuracy and later face recognition as they occlude facial information that is used to determine a face’s age and identity. To establish whether this is the case, we had participants estimate the age of unfamiliar faces that were pictured wearing no disguises, sunglasses, or face masks. The participants then completed a face recognition test where they had to distinguish between the previously seen faces and new faces. Importantly, none of faces wore disguises in this latter test. Participants’ estimates of the undisguised faces’ ages were inaccurate by a Median of 5.15 years. Their accuracy barely changed when the faces wore sunglasses but declined by a Median of 1.30 years when they wore face masks. Moreover, subsequent undisguised face recognition was less likely to occur when the faces previously wore sunglasses or face masks, with large effects observed. These findings demonstrate the relative importance of different facial areas when estimating faces’ ages and later recognising them. They also have implications for policing as they suggest it may be harder for eyewitnesses to accurately estimate the age of criminals who wear face masks during offences, and it may be harder for them to later recognise criminals in line-ups if the criminals wear sunglasses or face masks during offences.

## Significance statement

When an eyewitness sees a stranger commit a crime, the police often ask the eyewitness to estimate that stranger’s age. The police then focus their investigation on identifying a similarly aged suspect. If an eyewitness’s age estimate is inaccurate, the police can waste time/resources investigating innocent people. If a suspect is identified, the eyewitness may be shown their photograph in a line-up and asked if they were the criminal. If a criminal is not recognised, a crime can remain unsolved. Criminals sometimes disguise their appearance during offences with sunglasses or face masks that cover the lower half of their face. It is therefore important to know whether these disguises impact people’s ability to accurately estimate their age and later recognise them when undisguised. In theory, both disguises should make both tasks harder, as the eye region and lower half of the face contain important information about a person’s age and identity. We found estimates of undisguised and unfamiliar faces’ ages were inaccurate by a Median of 5.15 years. Accuracy barely changed when the faces wore sunglasses but declined by a Median of 1.30 years when they wore face masks. Moreover, undisguised faces were less likely to be recognised if previously seen wearing sunglasses or face masks. Thus, it may be harder for eyewitnesses to accurately estimate the age of unfamiliar criminals who wear face masks during offences, and it may be harder for them to recognise unfamiliar criminals in line-ups if the criminals wear sunglasses or face masks during offences.

## Introduction

Age estimation and face recognition are important in many criminal investigations. If you saw a stranger commit a crime and reported it to the police, the police would likely ask you to estimate that stranger’s age. The police would then focus their investigation on identifying a similarly aged suspect (Thorley et al., 2018). If your age estimate was inaccurate, the police could waste time and resources investigating innocent people. If a suspect was identified, you could be shown their photograph in a line-up and asked if they were the criminal (Brewer & Doyle, 2021). If the suspect was the criminal but you did not recognise them, the crime could remain unsolved. Alternatively, if you mistakenly identified an innocent suspect as the criminal, a miscarriage of justice could occur (Innocence Project, 2020). It is therefore important to know how accurately you could estimate the stranger’s age, how likely it is you would later recognise them, and what forensically relevant factors may impair these abilities.

Criminals sometimes disguise their facial features during offences by wearing sunglasses or face masks that cover the lower half of their face (e.g. Kossoff, 2020; Southall & Van Syckle, 2020; van Koppen & Lochun, 1997). Indeed, during the current COVID-19 pandemic, when many governments have required their citizens to wear face masks in public, there has been an increase in the number of criminals wearing face masks during offences (e.g. Rawlinson, 2021; Ward, 2020). Criminals are presumably doing this in the hope that it will stop eyewitnesses being able to provide a complete description of them to the police and/or identify them in a later line-up. Currently, little is known about the relative impact of sunglasses and face masks on our ability to accurately estimate strangers’ ages and later recognise them when undisguised. It is, however, established that the facial features occluded by sunglasses and face masks are sometimes used to estimate strangers’ ages and recognise them on subsequent encounters (Bruce & Young, 1986; Rhodes, 2009). The current study therefore examined whether estimates of unfamiliar faces’ ages are less accurate if they are wearing sunglasses or face masks and whether these disguises make it harder to later recognise those faces when undisguised.

### Age estimation, sunglasses, and face masks

How do we estimate an unfamiliar face’s age? A person’s face changes in a number of predictable ways with age and age estimates are largely based on the extent to which these changes have occurred (Coleman & Grover, 2006; Ko et al., 2017; Lam, 2015; Rhodes, 2009). For example, a face’s shape changes with age. Young adults typically have full cheeks and a well-defined jawline, giving their faces a V-shaped appearance. As adults enter middle age, however, their cheeks lose their fullness and gravity/soft tissue laxity creates jowls, giving their faces a U-shaped appearance. A face’s internal features also change with age. For example, fatty tissue loss around the eyes gives them an increasingly sunken appearance, gravity causes the nose and earlobes to slowly elongate, and facial bone density loss causes the lips to gradually roll inward and appear thin. Additionally, a face’s skin changes with age as wrinkles and an uneven skin tone develop. Doctoring images of faces to increase (decrease) these signs of ageing can increase (decrease) a face’s perceived age (e.g. Aznar-Casanova et al., 2010; Burt & Perrett, 1995; Fink & Matts, 2008; George & Hole, 1998; Porcheron et al., 2014).

Converging evidence suggests that when we estimate unfamiliar faces’ ages, we focus on their central facial region, which consists of their eyes, nose, and lips. For example, Liao et al. (2020) tracked participants’ eye movements when estimating the age of unfamiliar female faces and found they spent most time looking at the faces’ eyes (2477 ms), nose (1537 ms), and mouth (724 ms). Far less time was spent looking at other areas, such as their neck (173 ms). Moreover, the strongest predictors of an unfamiliar face’s perceived age come from its central facial region, such as its eye wrinkle depth, nasolabial fold wrinkle depth, and lip volume (Gunn et al., 2009; Merinville et al., 2015; Nkengne et al., 2008).^1^

Given the face’s importance in determining someone’s age, age estimation accuracy is typically studied by presenting participants with passport-style photographs of unfamiliar faces and asking them to estimate the faces' ages in years. Estimates of unfamiliar faces’ ages are often inaccurate by an average of approximately 6 years. For example, Voelkle et al. (2012) found 20–81-year-old participants’ estimates of 19–80-year-old strangers’ ages were inaccurate by an average of 6.35 years. Moreover, those same authors found participants overestimated young adults’ ages, underestimated elderly adults’ ages, and had more variable estimates of middle-aged adults’ ages (see also Short et al., 2019; Vestlund et al., 2009; Willner & Rowe, 2001). Additionally, Voelkle et al. found that age estimates are prone to an own-age bias, meaning estimates of unfamiliar faces’ ages are most accurate when the faces come from our own-age group (see also George & Hole, 1995; Klugman, 1947; Moyse & Brédart, 2012).

Thorley (2021) recently examined whether estimates of unfamiliar faces’ ages are less accurate when their eye region is disguised by sunglasses. To do this, he had young adults estimate the age of 20–85-year-olds who were wearing no disguise or sunglasses. Age estimates were most accurate when the faces belonged to people in their twenties and the degree of inaccuracy observed was equivalent regardless of whether they were wearing sunglasses or not (*M* inaccuracy = 5.32 years and 5.10 years, respectively). Age estimates became less accurate as the faces’ chronological ages increased (i.e. an own-age bias was evident) and the inaccuracy was exacerbated when the faces were wearing sunglasses. Thus, whether or not estimates of unfamiliar faces’ ages are less accurate when they are wearing sunglasses may depend upon the relative age of the estimator and the face whose age is being estimated.

To date, no studies have examined whether estimates of unfamiliar faces’ ages are less accurate when the lower half of the face is disguised by a face mask. In the closest approximation of this, Hole and George (2011) had young adult participants estimate the age of unfamiliar faces that had their whole face visible or only the upper half of their face visible due to the lower half being digitally erased. Whilst Hole and George did not compare age estimation accuracy across both conditions, results in their Table 1 shows that age estimates were inaccurate by an average of 1.6 years when whole faces were visible and 3.2 years when the lower half of faces was erased. Thus, digitally erasing facial information that would also be occluded by a face mask impaired age estimation accuracy.

### Face recognition, sunglasses, and face masks

Multiple studies show that familiar face recognition and unfamiliar face recognition (i.e. recognition of once-seen strangers’ faces) differ in important ways. For example, we are better at recognising familiar faces than unfamiliar faces (e.g. Chapman et al., 2018; Ellis et al., 1979; Klatzky & Forrest, 1984; Yarmey, 1971). Moreover, changes in expression, lighting, and viewpoint do not generally harm our ability to recognise familiar faces but can harm our ability to recognise unfamiliar faces (e.g. Hill & Bruce, 1996; O'Toole et al., 1998; Patterson & Baddeley, 1977). For brevity, we will discuss past research on unfamiliar face recognition only as that is most relevant to our current study.

How do we recognise unfamiliar faces? The exact mechanisms involved in learning and recognising unfamiliar faces are still being determined. Individual faces do, however, differ in a number of ways, including the size and shape of their facial features (e.g. round vs. almond eyes; hooked vs. button noses), the relative positions of these features (e.g. wide vs. close set eyes), their skin texture (e.g. smooth vs. rough), and their hairstyles. Importantly, there is evidence that we can differentiate unfamiliar faces via one or more of these defining characteristics (e.g. Cabeza & Kato, 2000; Ellis et al., 1979; Itz et al., 2017). This process, however, is complicated by the fact that these defining characteristics can appear different each time a face is encountered. For example, a person’s eye shape can change with their expression, whilst the distance between their eyes can appear to change when their face is viewed from a novel angle (e.g. see Burton et al., 2015, for a demonstration with familiar faces). Unfamiliar face recognition theories therefore need to account for this variation across encounters (e.g. see Turk & Pentland, 1991, for one such theory).

Several studies have examined whether it becomes harder to recognise undisguised faces if they were initially seen wearing sunglasses, relative to no sunglasses. For example, in a study similar to ours, Nguyen and Pezdek (2017) presented participants with photographs of unfamiliar faces that were wearing no disguise or sunglasses. The participants then completed a face recognition test where they had to distinguish between the previously seen faces and new faces, all of whom were undisguised. The participants were less likely to recognise the faces previously seen wearing sunglasses (see also Mansour et al., 2020). Separately, face matching studies show participants have difficulty determining whether two different photographs depict the same or different unfamiliar faces when the faces depicted are wearing sunglasses in one photograph or both, relative to no sunglasses in either (Graham & Ritchie, 2019; Noyes et al., 2021). Combined, these findings suggest we have difficulty processing and later recognising unfamiliar faces if they are wearing sunglasses.

To our best knowledge, no studies have yet examined whether it is harder to recognise undisguised faces if they were previously seen wearing surgical-style face masks, relative to no face masks. However, the aforementioned Nguyen and Pezdek (2017) study also found participants had difficulty recognising undisguised faces if they were previously seen wearing a bandana that covered the lower half of their face, relative to no disguise. Interestingly, those same authors also found participants had greater difficulty recognising undisguised faces if they had previously been seen wearing sunglasses, relative to a face bandana. They suggest this may have occurred due to information from the eye region being more important than information from the mouth region when differentiating unfamiliar faces (Davies et al., 1977; McKelvie, 1976). Separately, Freud et al. (2020) found participants had difficulty recognising unfamiliar face-masked faces on subsequent encounters when the faces always wore a face mask. Additionally, face matching studies show participants have greater difficulty determining whether two different photographs depict the same or different unfamiliar faces when the faces are wearing a face mask in one photograph or both (e.g. Carragher & Hancock, 2020; Noyes et al., 2021). Combined, these findings suggest we also have difficulty processing and later recognising unfamiliar faces if they are wearing face masks.

It is perhaps unsurprising that unfamiliar faces which are initially seen wearing sunglasses or face masks are harder to recognise when later seen undisguised, as both disguises would stop participants encoding the unique facial characteristics that would later help them differentiate the previously seen faces from new faces. Nguyen and Pezdek’s (2017) observation that sunglasses impair later undisguised face recognition more than a face bandana, however, suggests different disguises may impair later undisguised face recognition to different extents. This latter issue warrants further investigation to determine its generalisability.

### Aims and hypotheses

This study had two aims. The first was to examine whether or not it is harder to accurately estimate the age of unfamiliar young adults’ faces when they are wearing sunglasses or a face mask, relative to when they are undisguised. The second was to examine whether or not it is harder to recognise unfamiliar/undisguised young adults’ faces if they were previously seen wearing sunglasses or a face mask, relative to no disguise. To examine these issues, we had participants estimate the age of unfamiliar young adults’ faces that were pictured wearing no disguise, sunglasses, or a face mask. The participants then completed a face recognition test where they had to discriminate between the previously seen faces and new faces. Importantly, none of the faces were wearing sunglasses or face masks during the face recognition test.

All faces in this study belonged to young adults as we wished to test the replicability of Thorley’s (2021) previous finding showing estimates of their ages are no less accurate when they are pictured wearing sunglasses. His finding could be considered surprising, given that the eye region contains important information about a face’s age. If his finding replicates with new participants and faces, this suggests the eye region does not contain critical information about a young adult’s age and that information from other facial areas is as informative when their eye region is occluded. One of those other areas could be the lower half of their face. Ours is the first study to examine whether estimates of young adults’ ages are less accurate when the lower half of their face is occluded by a face mask. Given that the nose and mouth areas also contain important information about a face’s age, it is tentatively expected that estimates of the young adults’ ages will be less accurate if they are wearing a face mask. Importantly, if estimates of their ages are no less accurate when they are wearing sunglasses but are less accurate when they are wearing a face mask, this would provide initial evidence that the lower half of a young adult’s face contains more important information about their age than their eye region.

Focussing on face recognition, past research suggests our participants will have greater difficulty recognising undisguised faces that were previously seen wearing sunglasses, relative to no disguise (e.g. Mansour et al., 2020; Nguyen & Pezdek, 2017). Whilst no studies have previously examined whether it is harder to recognise undisguised faces that were previously seen wearing a face mask, relative to no disguise, one study has found it is harder to recognise undisguised faces if they were previously seen wearing a face bandana which covered some of the same facial areas as a face mask (Nguyen & Pezdek, 2017). Moreover, that same study found a face bandana was less disruptive to subsequent face recognition than sunglasses. Consequently, it is expected that our participants will have difficulty recognising undisguised faces that were previously seen wearing a face mask, but the effect will be smaller than when they were previously seen wearing sunglasses.

## Method

### Participants

Our ideal sample size was determined via a power analysis conducted in G*Power 3 (Faul et al., 2007). To recap, one goal of our study was to test the replicability of Thorley’s (2021) observation that participants’ estimates of young adults’ ages do not change when they are wearing sunglasses. Thorley did not report the size of his non-significant effect, but a re-examination of his data shows it was small (*d* = 0.06). It was therefore critical our analyses could detect small effects. The power analysis indicated 163 participants would be required to detect a small effect with the one-way repeated-measures ANOVA we anticipated using in all analyses (Cohen’s *f* = 0.10; 1 − *β* = 0.80, *α* = 0.05). To maintain sufficient Power if any data had to be excluded, we aimed to recruit a minimum of 180 participants.

Our final sample had 230 participants. They were aged 17–60 (*M*_age_ = 21.79, *SD*_age_ = 6.49, Female = 169, Male = 59, Other = 2). All were studying psychology courses at a multi-campus university, were recruited via an online sign-up system, and received course credit for participation. We excluded seven additional participants who started the study but did not finish it and two additional participants who made the same responses on ≥ 95% of the face recognition test trials.

### Design

This study had one repeated-measures independent variable (Disguise Type) with three levels (no disguise, sunglasses, or face mask).

There were two age estimation measures. These were age estimation accuracy and age estimation bias. *Age estimation accuracy* is an absolute measure of how accurate age estimations are. It is calculated by subtracting each age estimate (e.g. 25 years) from each face's chronological age (e.g. 30 years) and determining the error (e.g. 5 years). Each participant’s errors are then averaged, producing their Mean age estimation accuracy score. Importantly, during the calculations, negative values from underestimations are treated as positive values. This stops underestimations and overestimations cancelling each other out during the averaging process and producing overly conservative Mean scores. *Age estimation bias* is a measure of whether participants tended to underestimate or overestimate faces' ages. It is calculated in a near identical way to age estimation accuracy, except underestimations are treated as negative values during the averaging process.

There were also two face recognition test measures. These were the proportion of *hits* and *false alarms* participants made when trying to discriminate faces whose ages they had estimated (henceforth called targets) from new faces whose ages they had not estimated (henceforth called fillers). A hit occurred when a participant correctly recognised a target as a target. A false alarm occurred when a participant incorrectly recognised a filler as a target. The hit and false alarm rates were then used to calculate signal detection theory (SDT) measures called *discrimination* and *response bias* (see Stanislaw & Todorov, 1999, for a detailed overview of SDT and both measures’ calculations). Here, discrimination is a measure of participants’ ability to distinguish targets from fillers. Response bias is a measure of whether participants had a bias towards classing recognition test faces as targets (i.e. a liberal response bias) or fillers (i.e. a conservative response bias).

### Stimuli

During the age estimation task, participants saw passport-style colour photographs of 30 unfamiliar White adult faces from the Center for Vital Longevity Face Database (Minear & Park, 2004). Half of the faces were female, half male, and they were aged 18–32 (*M*_age_ = 22.83, *SD*_age_ = 3.24). All faces had a neutral expression and appeared against a neutral grey background. None had facial accessories (e.g. glasses).

**Fig. 1:**
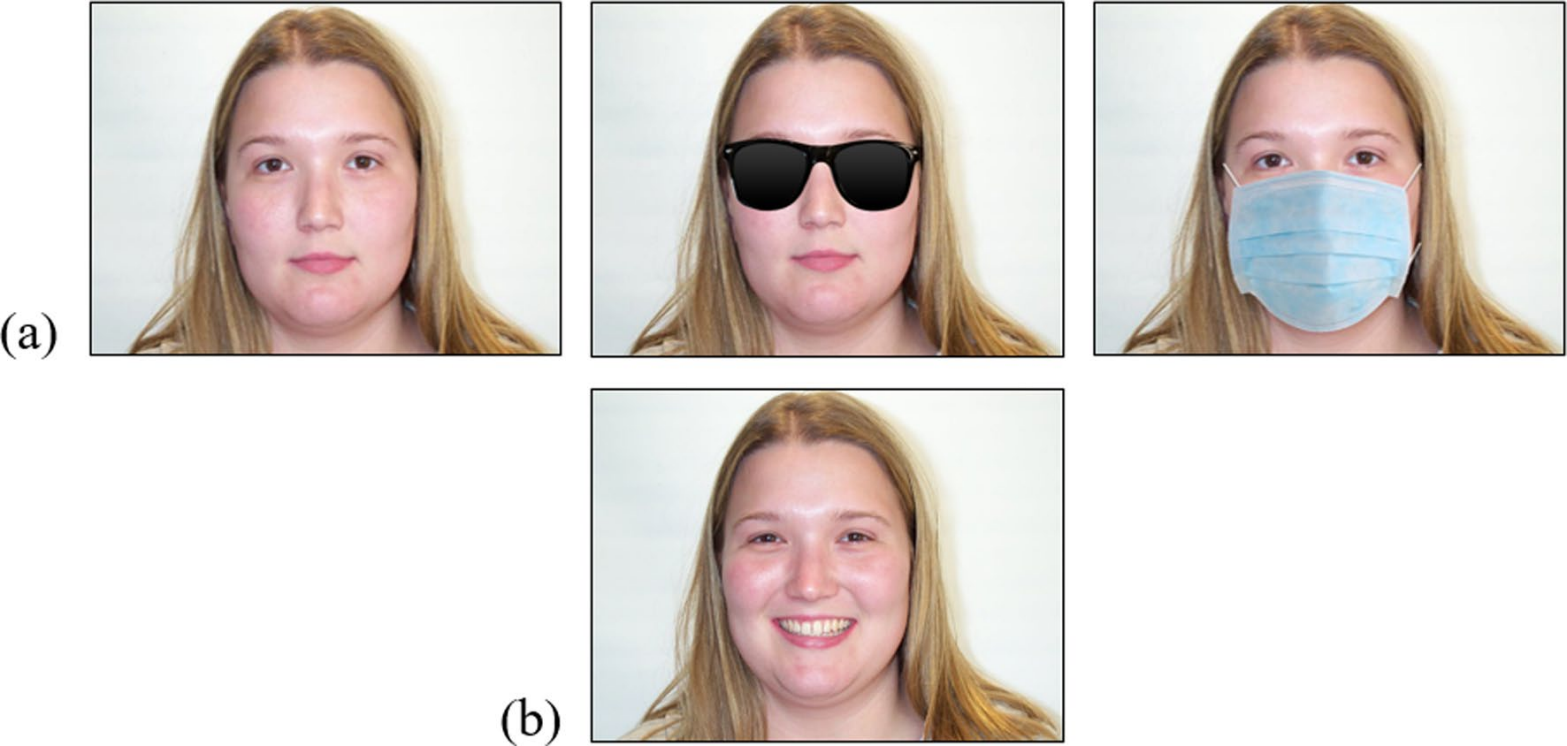
Example stimuli from the age estimation task and face recognition test. *Note*: Row (**a**) depicts one of the faces from the age estimation task that was shown wearing either no disguise, sunglasses, or a face mask. The woman pictured is 23 years old. Row (**b**) depicts the alternative image of this woman that was used during the face recognition test

All 30 photographs were digitally edited so two new versions were created. In one new version, sunglasses were added to the faces. The sunglasses were black and opaque, so no information from within the faces’ eye region was visible (although the eyebrows were visible). In the other new version, a surgical face mask was added to the faces. The face masks always covered the lower half of the face, from the chin to near the top of the nose. See Fig. 1a for examples.

During the face recognition test, participants saw alternative photographs of the 30 faces from the age estimation task (i.e. the targets). In these photographs, each face was now smiling and none were wearing sunglasses or a face mask. See Fig. 1b for an example. During the face recognition test, the participants also saw photographs of 30 new faces (i.e. the fillers). These photographs were also taken from the Center for Vital Longevity Face Database and had similar characteristics to the targets (e.g. 15 males, 15 females, age range = 19–34, *M*_age_ = 22.73, *SD*_age_ = 3.15, all White, all smiling).

### Procedure

The study was hosted online using Qualtrics. Participants first read an information page stating that the study was investigating their ability to estimate strangers' ages and that some strangers may be wearing sunglasses or face masks. The subsequent face recognition test was not mentioned. This page also asked participants to complete the study individually, in a distraction-free environment, on a device of their choosing. After reading the information page and consenting to participate, the participants completed a demographic questionnaire. They were then presented with the 30 target faces. Each face was presented individually and once only, in a fully randomised order, for 5 s. Participants always saw 10 faces without any disguise (5 male, 5 female), 10 with sunglasses (5 male, 5 female), and 10 with a face mask (5 male, 5 female). The faces in each disguise condition were counterbalanced across participants (e.g. Participant 1 saw a 22-year-old female without a disguise, Participant 2 saw her with sunglasses, and Participant 3 her with a face mask). After seeing a face for 5 s, the face disappeared. Participants were then asked to estimate that face’s age in years. An estimate had to be provided before seeing the next face and that estimate could only be a two-figure numerical value (e.g. 25).

After estimating all 30 faces’ ages, participants completed the face recognition test. Prior to commencing this test, onscreen instructions stated “*You will now be asked to complete one final task. Next you will see 60 people. You will have previously estimated the age of some of these people. Please indicate whether you previously estimated the age of each person or not*”. The face recognition test then started. During this test, the participants saw the alternative photographs of the 30 faces from the age estimation task (i.e. the targets) and the photographs of the 30 fillers. Each face was presented individually, and once only, in a fully randomised order. Alongside each face was the question “*Did you estimate the age of this person?*” and a “*yes/no*” response option. Each face remained onscreen until participants responded. Once participants responded, the next face appeared. After responding to all 60 faces, the participants read a debrief page and the study ended. On average, the study took 12 min to complete.

## Results

The data analysed in this study can be downloaded from the OSF repository at https://osf.io/9xdn5/?view_only=8aeee55a0c0045f48596e3eeb3fd29b4.

### Age estimation accuracy

To recap, age estimation accuracy is an absolute measure of how accurate age estimates are. The Mean and Median age estimation accuracy rates in the three disguise conditions, and associated standard deviations, are in Table 1. Across all conditions, age estimates were inaccurate by an average of 5.92 years (*Mdn* = 5.50, *SD* = 2.40). Ordinarily, a parametric one-way repeated-measures ANOVA would be used to compare the accuracy rates within each disguise condition. However, Shapiro–Wilk tests suggested the accuracy scores within each condition had non-normal distributions (*p*’s < 0.05) and histograms showed they were positively skewed. Consequently, a parametric test could not be used. Instead, the accuracy rates within each condition were compared via a nonparametric Friedman’s ANOVA. For each Friedman’s ANOVA in this article, Kendall’s *W* was used as an effect size measure (0.1–< 0.3 = a small effect, 0.3–< 0.5 = a moderate effect, > 0.5 = a large effect).

**Table 1:** Mean and median age estimation accuracy and bias, in years, in the three disguise conditions

	No disguise	Sunglasses	Face mask
*Age estimation accuracy*			
Mean	5.44 (2.16)	5.62 (2.31)	6.70 (2.54)
Median	5.15	5.20	6.45
*Age estimation bias*			
Mean	4.44 (2.78)	4.61 (2.91)	5.72 (3.09)
Median	4.30	4.40	5.70

Standard deviations are in parentheses

The Friedman’s ANOVA identified a statistically significant difference in the age estimation accuracy rates when the faces wore no disguise, sunglasses, or a face mask, ${\chi }_{w}^{2}$(2) = 70.43, *p* < 0.001, *W* = 0.70. Three Conover multiple comparison tests were then conducted comparing the age estimation accuracy rates in each disguise condition. For all multiple comparisons in this article, a Holm correction was applied to the observed *p* values. That correction meant an alpha of 0.05 could be retained as the cut-off point for statistical significance. The Conover tests showed age estimation accuracy did not significantly differ when the faces wore no disguise or sunglasses (*p* = 0.34, *r* = 0.09). Age estimates were, however, less accurate when the faces wore a face mask, relative to no disguise or sunglasses (both *p*'s < 0.001, *r*’s = 0.60 and 0.55, respectively). It is, therefore, harder to accurately estimate a face’s age if it is wearing a face mask.

### Age estimation bias

To recap, age estimation bias is a measure of whether participants tended to underestimate or overestimate faces' ages. Regardless of whether the faces wore a disguise or not, participants overestimated their ages (see Table 1). We initially examined whether or not the degree of bias observed in each disguise condition was significantly different from zero. This was done using one-sample *t* tests in the no-disguise and face mask conditions. A one-sample Wilcoxon signed-rank test was used in the sunglasses condition, as a Shapiro–Wilk test suggested the data in this condition had a non-normal distribution (*p* < 0.05). These tests showed that the degree of bias was significantly different from zero in the no disguise condition, *t*(229) = 24.16, *p* < 0.001, *d* = 1.59, sunglasses condition, *T*(229) = 26,302.50, *p* < 0.001, *r* = 0.98, and face mask condition, *t*(229) = 28.13, *p* < 0.001, *d* = 1.86.

Next, a Friedman’s ANOVA was used to compare the degree of bias in each disguise condition. That test identified a statistically significant difference in age estimation bias when the faces wore no disguise, sunglasses, or a face mask, ${\chi }_{w}^{2}$(2) = 52.39, *p* < 0.001, *W* = 0.72. Conover tests revealed no statistically significant difference in the degree of overestimation when the faces wore no disguise or sunglasses (*p* = 0.74, *r* = 0.06), but greater overestimation when the faces wore a face mask, relative to no disguise (*p* < 0.001, *r* = 0.50) or sunglasses (*p* < 0.001, *r* = 0.48). Face masks therefore made young adults appear older than they are.

### Face recognition

As mentioned, prior to calculating the two SDT measures (discriminability and response bias) it was necessary to calculate the proportion of hits and false alarms in each disguise condition. To recap, a hit occurred when a participant correctly recognised one of the 30 targets as a face they had estimated the age of. A false alarm occurred when they incorrectly recognised one of the 30 fillers as a face they had estimated the age of. Each participant had three Mean hit scores. The first was derived from their responses to the ten targets in the no disguise condition, the second was derived from their responses to the ten targets in the sunglasses condition, and the third was derived from their responses to the ten targets in the face mask condition. Each participant, however, had only one Mean false alarm score, which was derived from their responses to the 30 fillers. To calculate discriminability and response bias, participants needed to have Mean false alarm scores associated with each disguise condition (e.g. a Mean false alarm score associated with the no disguise condition, a Mean false alarm score associated with the sunglasses condition, and so on). We therefore split each participant’s 30 filler responses into three groups of ten. This was done using random sampling with replacement. For example, Participant 1’s responses to fillers 3, 4, 8, 10, 15, 17, 22, 23, 27, and 29 may have been randomly selected to form one group of ten. The same participant’s responses to ten other fillers would have then been randomly selected to form another group of ten, and so on. We then calculated the Mean false alarm scores for each group of ten. Once this had been done, each participant’s three Mean false alarm scores were randomly paired with one of their three Mean hit scores (e.g. one Mean false alarm score became paired with their Mean hit score in the no disguise condition, another became paired with their Mean hit score in the sunglasses condition, and so on). These pairings were then used to calculate the discrimination and response bias measures.

Prior to calculating the discrimination and response bias measures, data screening was conducted. Shapiro–Wilk tests suggested the hit and false alarm scores associated with each disguise condition had non-normal distributions (*p*’s < 0.001). Histograms confirmed the hit scores were negatively skewed and the false alarm scores were positively skewed. Consequently, the hits and false alarms were used to calculate nonparametric measures of discriminability and response bias called *A*′ and *B*″, respectively. *A*′ typically ranges from 0.5 (indicating targets cannot be distinguished from fillers) to 1 (indicating perfect discrimination). Values less than 0.5 may arise from sampling error or response confusion; 0 is the lowest possible value. *B*″ ranges from − 1 (an extreme liberal response bias) to 1 (an extreme conservative response bias). A value of 0 signifies no response bias. The Mean hits, false alarms, *A*′ scores, *B*″ scores, and associated standard deviations, per disguise condition are in Table 2.

**Table 2:** Mean hits, false alarms (FA’s), discrimination (*A*′), and response bias (*B*″) on the face recognition test across the three disguise conditions.

	No disguise	Sunglasses	Face mask
Hits	0.81 (0.16)	0.62 (0.19)	0.66 (0.19)
FA’s	0.23 (0.19)	0.22 (0.17)	0.23 (0.17)
*A*′	0.86 (0.11)	0.78 (0.13)	0.79 (0.12)
*B*″	0.01 (0.64)	0.27 (0.47)	0.24 (0.51)

The hit and false alarm values are proportions

Standard deviations are in parentheses

Participants’ ability to discriminate targets from fillers in the three disguise conditions, as indicated by their *A′* scores, was compared using a Friedman’s ANOVA. There was a statistically significant difference in their discriminability across the three disguise conditions, ${\chi }_{w}^{2}$(2) = 90.64, *p* < 0.001, *W* = 0.57. Conover tests showed participants were worse at discriminating targets from fillers when the targets had previously worn sunglasses (*Mdn* = 0.81) or a face mask (*Mdn* = 0.83), relative to no disguise (*Mdn* = 0.88; both *p*’s < 0.001, *r*’s = 0.67 and 0.62, respectively). There was no significant difference in their ability to discriminate targets from fillers when the targets wore either disguise type (*p* = 0.29, *r* = 0.11). It is, therefore, harder to distinguish once-seen seen faces from new faces when the once-seen faces were previously wearing sunglasses or a face mask, relative to no disguise.

Overall, participants had no response bias when targets were originally seen undisguised but had a conservative response bias when targets previously wore disguises. A Friedman’s ANOVA showed a statistically significant difference in participants’ response bias across the three disguise conditions when trying to discriminate targets from fillers, ${\chi }_{w}^{2}$(2) = 37.86, *p* < 0.001, *W* = 0.57. Conover tests confirmed participants were more likely to class faces as fillers on the face recognition test when they had previously seen targets wearing sunglasses (*Mdn* = 0.20) or a face mask (*Mdn* = 0.14), relative to no disguise (*Mdn* = 0.00; both *p*'s < 0.001, *r*’s = 0.49 and 0.42, respectively). There was no significant difference in their response bias when the targets previously wore either type of disguise (*p* = 0.26, *r* = 0.07). In sum, participants became reluctant to say they recognised undisguised faces when those faces were previously seen wearing sunglasses or a face mask.

## General discussion

This study had two aims. The first was to examine whether or not it is harder to accurately estimate the age of unfamiliar young adults’ faces when they are wearing sunglasses or a face mask, relative to when they are undisguised. Here, estimates of their ages were less accurate when they wore face masks but not when they wore sunglasses. The second aim was to examine whether or not it is harder to recognise unfamiliar/undisguised young adults’ faces if they were previously seen wearing sunglasses or a face mask, relative to no disguise. Here, both disguises harmed later undisguised face recognition.

We will now discuss our age estimation results in more detail. Consistent with past research, our participants overestimated the undisguised young adults’ ages (e.g. see Short et al., 2019; Vestlund et al., 2009; Voelkle et al., 2012; Willner & Rowe, 2001). Moreover, their estimates were inaccurate by a Median of 5.15 years. Studies similar to ours have also shown estimates of undisguised young adults’ ages are inaccurate by an average that is close to 5 years (e.g. Short et al., 2019; Thorley, 2021; Voelkle et al., 2012). Additionally, we replicated Thorley’s (2021) past result showing participants continue overestimating young adults’ ages when they are wearing sunglasses. Moreover, we replicated his past result showing participants’ age estimates are no less accurate in these circumstances. There is, therefore, converging evidence that estimates of young adults’ ages do not change when they are wearing sunglasses. A new finding here is that our participants continued overestimating the young adults’ ages when they wore face masks. Moreover, we found their estimates became less accurate in these circumstances. More specifically, their perceived age increased by a Median of 1.30 years if they wore a face mask. Thus, our results suggest that face masks make unfamiliar young adults appear older than they are.

Our findings offer an insight into the relative importance of different facial areas when estimating unfamiliar young adults’ ages. Past research has already shown that when estimating unfamiliar faces’ ages, we focus on their eyes, nose, and mouth areas (Liao et al., 2020). Moreover, the strongest predictors of a face’s likely age come from cues in those areas, such as its eye wrinkle depth, nasolabial fold wrinkle depth, and lip volume (Gunn et al., 2009; Merinville et al., 2015; Nkengne et al., 2008). Thus, whilst we may use information from the eye region to estimate a young adult’s age when it is available, our findings, alongside Thorley’s (2021), suggest the eye region may not contain critical information about a young adult’s age, as age estimation accuracy does not decline if that region is occluded by sunglasses. Instead, our findings suggest the lower half of the face may contain critical information about a young adult’s age, as age estimation accuracy declines if that region is occluded by a face mask. It remains to be determined why face masks harmed age estimation accuracy in our study but sunglasses did not. One potential explanation is that face masks hide a greater amount of facial information than sunglasses, meaning they occlude a larger number of ageing cues (e.g. nasolabial fold wrinkle depth, lip volume, lower face skin texture).

Whilst Thorley (2021) and the current study both showed estimates of young adults’ ages are no less accurate when they wear sunglasses, Thorley did find that estimates of elderly adults’ ages were less accurate when they wore sunglasses. Combined, these findings suggest that the facial regions used to estimate faces’ ages may vary in importance as their chronological age changes. Future studies may wish to investigate this possibility.

Focussing on our face recognition results, undisguised/unfamiliar young adult faces were less likely to be recognised when they had previously been seen wearing sunglasses or a face mask, relative to no disguise. Similar impairments have been observed in past studies when unfamiliar/undisguised faces were initially seen wearing sunglasses or a face bandana (Mansour et al., 2020; Nguyen & Pezdek, 2017). Our findings are perhaps unsurprising as we can differentiate unfamiliar faces via their defining characteristics, such as the size and shape of their facial features, the relative positions of these features, their skin texture, and their hairstyles (e.g. Cabeza & Kato, 2000; Ellis et al., 1979; Itz et al., 2017). If some of these characteristics are occluded by sunglasses or a face mask when faces are initially encountered, they cannot be encoded and stored in memory. This would then make it harder to later recognise those faces when undisguised. Our findings are complimented by those showing we have difficulty recognising face-masked faces when they are repeatedly seen wearing a mask (Freud et al., 2020). They are also complimented by those from studies showing we have difficulty determining whether two different photographs depict the same or different faces when the faces are wearing sunglasses or a face mask in one or both pictures (e.g. Carragher & Hancock, 2020; Noyes et al., 2021). Combined, these findings all suggest that sunglasses and face masks cause a general face perception deficit.

Interestingly, participants had no response bias during the face recognition test when deciding whether previously undisguised faces had appeared during the age estimation task. They did, however, have a conservative response bias when deciding whether previously disguised faces had appeared during that task, with the response bias equivalent regardless of the disguise worn. Thus, the disguises made participants reluctant to class previously disguised faces as recognised. Our findings compliment those from face matching studies by Carragher and colleagues, which found participants become reluctant to declare that two different photographs of the same face show the same person when that person is wearing a face mask in one photograph (Carragher & Hancock, 2020; Carragher et al., 2021).

Previously, Nguyen and Pezdek (2017) found that sunglasses impaired subsequent undisguised face recognition more than a face bandana. They suggest this may have occurred due to information from the eye region being more important than information from the mouth region when differentiating unfamiliar faces (Davies et al., 1977; McKelvie, 1976). In our study, however, the sunglasses and face mask impaired subsequent undisguised face recognition to a similar extent, suggesting the facial areas occluded by both disguises contain equally important information about a stranger’s identity. Whilst it is not immediately obvious why our findings differ from those of Nguyen and Pezdek (2017), one possible reason is that our face mask occluded a larger proportion of the face than their bandana. More specifically, our face mask occluded facial features from the bottom of the chin to the top of the nose whilst their face bandana occluded facial features from the bottom of the chin to the bottom of the nose. It is therefore possible that the greater degree of facial occlusion by the face mask in our study made subsequent undisguised face recognition harder and its disruptive effects were on par with the sunglasses. This point is elaborated on in the next section.

## Limitations

Our study had several limitations that may impact the generalisability of its findings. The first is that participants estimated the age of faces in photographs and not “live” strangers. When we encounter strangers in everyday contexts, there can be additional cues signalling their age such as the sound of their voice and posture (Moyse, 2014; Rexbye & Povlsen, 2007). It is currently unknown whether or not these additional cues improve estimates of young adult strangers’ ages. Voelkle et al. (2012), however, had participants estimate the age of 19–80-year-old strangers’ faces in photographs and found their estimates were inaccurate by an average 6.35 years. In contrast, Amilon et al. (2007) had participants estimate the age of 17–81-year-old strangers who were talking in videos and found their estimates were inaccurate by an average 5.10 years. Estimates of strangers’ ages do, therefore, appear to be more accurate when additional cues signalling their age are available. Consequently, estimates of young adult strangers’ ages may be more precise in everyday contexts than our study.

A second limitation is that participants knew they would be required to estimate unfamiliar faces’ ages, meaning they likely did this whilst looking at the faces (despite the actual age estimate being input into Qualtrics after the face disappeared). If so, the age estimation task would have been a largely perceptual task. In some everyday scenarios, such as eyewitness testimony scenarios, age estimations are made days, weeks, or months after viewing a stranger’s face. In those instances, the age estimation switches from being a perceptual task to a memory task. Readers may therefore question whether our findings apply to everyday scenarios where age estimations are based on memory. In defence of our procedures, Ebbesen and Rienick (1998) found estimates of strangers’ ages were consistently accurate regardless of whether they were made whilst looking at the stranger in person, after 1 day, after 7 days, after 28 days, or at all four time points. This (admittedly limited) evidence, therefore, suggests our findings may generalise to scenarios where age estimations are based on memory.

A third limitation is that our participants encoded faces under idealised conditions. Everyday viewing conditions are often less ideal, making it harder to encode new faces and remember them (Hancock et al., 2000). Face recognition accuracy rates may, therefore, be lower in everyday contexts than in our study.

A fourth limitation of our study is that participants were predominantly young adults, whilst the unfamiliar faces all belonged to young adults. Age estimation accuracy and face recognition are both prone to own-age biases, meaning people are better at estimating the age of faces/later recognising those faces when they come from their own-age group (e.g. Anastasi & Rhodes, 2006; Voelkle et al., 2012). Age estimation and face recognition in our study may, therefore, have been a little worse if the faces were from more diverse age groups. It is also unclear whether age estimation and face recognition become disproportionally less accurate when faces from other age groups wear sunglasses or face masks, so future researchers may wish to investigate this (but see Thorley, 2021).

A final limitation of our study is that the faces only wore one type of sunglasses and face mask. Other sunglasses or face masks can occlude more (or less) of a face and disguises that occlude more (or less) of a face could potentially make age estimation accuracy and face recognition more (or less) accurate. Future research should, therefore, examine whether or not this is the case.

## Applied implications

Our findings may be of interest to police investigators who, for reasons discussed, need to know whether eyewitnesses to a crime will find it harder to accurately estimate the age of criminals during interviews/later recognise those criminals in line-ups if the criminals wore sunglasses or a face mask during the crime. Our results suggest eyewitnesses may overestimate young adult criminals’ ages and that the degree of overestimation may be no worse if the criminal wore sunglasses during the crime. The degree of overestimation may, however, increase slightly if the criminal wore a face mask. Moreover, our results suggest eyewitnesses may be less likely to identify a criminal in a line-up if the criminal was wearing sunglasses or a face mask during the crime. It is emphasised, however, that multiple studies replicating these effects with more ecologically valid stimuli are essential before generalising our findings to real crime scenarios.

Our findings may also be of interest to salespersons who are responsible for selling age restricted goods, such as alcohol and tobacco. Our findings suggest that staff could overestimate young adult customers' ages by close to 4.5 years if they are undisguised or wearing sunglasses and by close to 5.7 years if they are wearing a face mask. If so, this could result in underage sales. This therefore reinforces the need for salespersons to ask for a proof of age if customers are potentially underage, especially if they are wearing a face mask for public health reasons.

## Conclusion

Our results show that when we try estimating an unfamiliar young adult’s age, we will likely overestimate their age by several years. Moreover, our age estimation may be no less accurate if they are wearing sunglasses. The degree of overestimation may, however, increase if they are wearing a face mask. Additionally, if the young adult is wearing sunglasses or a face mask, this may make it harder to later recognise them when undisguised. From a theoretical perspective, this knowledge is useful as it demonstrates the relative importance of different facial areas when estimating a young adult’s age and later recognising them. From an applied perspective, this knowledge is important as it provides an insight into how an eyewitness's ability to estimate a criminal’s age during a police interview and later recognise that criminal in a line-up may be impacted if the criminal was wearing sunglasses or a face mask during the crime.

## Data Availability

The data analysed in this study can be downloaded from the OSF repository at https://osf.io/9xdn5/?view_only=8aeee55a0c0045f48596e3eeb3fd29b4.

## Footnotes

1 Far less is known about the importance of a person’s hair when estimating their age. Balding, hair thinning, and greying can occur in males and females as they age and premature changes to a person’s hair can result in over-estimations of their age (Bulpitt et al., 2001; Butler et al., 1998; Gunn et al., 2009). Interviews and eye-tracking studies, however, suggest that we focus more on a person’s internal facial features than their hair when estimating their age (Liao et al., 2020; Rexbye & Povlsen, 2007). Moreover, Thorley (2021), George and Hole (1995), and others have found that occluding a person’s hair does not impact age estimation accuracy. Combined, this all suggests that a person’s hair may be of secondary importance when estimating their age.
